# Assessment of desiccants and their instructions for use in rapid diagnostic tests

**DOI:** 10.1186/1475-2875-11-S1-P8

**Published:** 2012-10-15

**Authors:** Barbara Barbé, Philippe Gillet, Greet Beelaert, Katrien Fransen, Jan Jacobs

**Affiliations:** 1 Department of Clinical Sciences, Institute of Tropical Medicine, Antwerp, Belgium

## Background

Malaria rapid diagnostic tests (RDTs) are protected from humidity-caused degradation by the inclusion of a desiccant in the device packaging. The present study assesses malaria RDT products for the availability, type and design of desiccants and their information supplied in the instructions for use (IFU).

## Materials and methods

A panel of malaria RDTs was assessed for the desiccant type supplied in the device packaging and for the information mentioned in the IFU. The criteria used during this assessment were based on recommendations of the World Health Organization, the European Community, the European Chemicals Agency and own observations.

Desiccant sachets were defined as self-indicating (all beads coated with a humidity indicator that changes color upon saturation), partial-indicating (part of the beads coated) and non-indicating (none of the beads coated).

In addition, silica gel sachets containing a humidity indicator were individually assessed for humidity saturation indicated by color change and, in case of partial-indicating silica gels, for the presence or absence of indicating beads.

## Results

Fifty malaria RDT products from 25 manufacturers were assessed, of which 14 (28%) products were listed by the “Global Fund Quality Assurance Policy” and 31 (62%) were CE-marked. All but one of the products contained a desiccant, which was generally silica gel (47/50, 94%) supplied as a sachet enclosed in the device packaging.

Twenty (40%) RDT products (one with no desiccant and 19 with non-indicating desiccant) did not meet the WHO guidelines recommending to add a self- indicating desiccant to the RDT device packaging. However, where self- or partial-indicating silica gel was added (n = 22 and 8 respectively), the toxic cobalt dichloride was always used as humidity indicator.

Less than half (14/30, 47%) of the IFUs of RDT products with indicating desiccants mentioned to check the humidity saturation before using the test. Moreover none of the IFUs included information on properties, safety hazards and disposal of the desiccant.

For the criteria assessed here, no difference was observed for Global Fund-listed and CE marked RDT products compared to those which were not.

A total of 15,577 silica sachets from 16 malaria RDT products were visually inspected immediately after opening the device packaging. Color change indicating humidity saturation was observed for 8/16 RDT products, at a median incidence of 0.8% (range 0.05% - 4.6%) of sachets inspected.

In all RDTs with partial-indicating silica gel, sachets without any color indicating bead were found (median proportion 13.5% (0.6% - 17.8%) per product) and an additional light source was needed to be able to assess the color of the humidity indicator.

## Conclusions

The design of desiccants currently provided in RDTs shows several shortcomings. Improvements can be made regarding desiccant type (self-indicating desiccant which is easy to inspect), desiccant safety (phasing out of cobalt dichloride) and information supplied in the IFU.

